# Frameworks, Models, and Theories Used in Electronic Health Research and Development to Support Self-Management of Cardiovascular Diseases Through Remote Monitoring Technologies: Protocol for a Metaethnography Review

**DOI:** 10.2196/13334

**Published:** 2019-07-16

**Authors:** Roberto Rafael Cruz-Martínez, Peter Daniel Noort, Rikke Aune Asbjørnsen, Johan Magnus van Niekerk, Jobke Wentzel, Robbert Sanderman, Lisette van Gemert-Pijnen

**Affiliations:** 1 Department of Psychology, Health and Technology Faculty of Behavioural, Management and Social sciences, Technical Medical Centre University of Twente Enschede Netherlands; 2 Embedded Information Services Library, ICT Services & Archive University of Twente Enschede Netherlands; 3 Research and Innovation Department Vestfold Hospital Trust Tønsberg Norway; 4 Saxion University of Applied Sciences Deventer Netherlands; 5 GZW-Health Psychology–GZW-General University Medical Center Groningen University of Groningen Groningen Netherlands

**Keywords:** eHealth, telemedicine, research and development, implementation, evaluation, multidisciplinary approach, meta-ethnography, systematic review, remote monitoring, self-management, cardiovascular diseases

## Abstract

**Background:**

Electronic health (eHealth) is a multidisciplinary and rapidly evolving field, and thus requires research focused on knowledge accumulation, curation, and translation. Cardiovascular diseases constitute a global health care crisis in which eHealth can provide novel solutions to improve the efficiency and reach of self-management support for patients where they most need it: their homes and communities. A holistic understanding of eHealth projects focused on such case is required to bridge the multidisciplinary gap formed by the wide range of aims and approaches taken by the various disciplines involved.

**Objective:**

The primary objective of this review is to facilitate a holistic interpretation of eHealth projects aimed at providing self-management support of cardiovascular diseases in the natural setting of patients, thus priming the use of remote monitoring technologies. The review aims to synthesize the operationalization of frameworks, models, and theories applied to the research and development process of eHealth.

**Methods:**

We will use Noblit and Hare’s metaethnography approach to review and synthesize researchers’ and practitioners’ reports on how they applied frameworks, models, and theories in their projects. We will systematically search the literature in 7 databases: Scopus, Web of Science, EMBASE, CINAHL, PsycINFO, ACM Digital Library, and the Cochrane Library. We will thoroughly read and code selected studies to extract both raw and contextual data for the synthesis. The relation of the studies will be determined according to the elements of the frameworks, models, or theories the studies applied. We will translate these elements between each other and intend to synthesize holistic principles for eHealth development for the case at hand.

**Results:**

The search strategy has been completed, data extraction is almost finalized, and the first synthesis approaches are underway. The search yielded 1224 citations and, after we applied the selection criteria, 17 articles remained. We expect to submit the final results for publication in 2019.

**Conclusions:**

This review is important because it aims to create a holistic understanding of a multidisciplinary topic at the crossroads of eHealth, cardiovascular diseases, and self-management. The value of metaethnography in contrast to other systematic review methods is that its synthesis approach seeks to generate a new understanding of a topic, while preserving the social and theoretical contexts in which findings emerge. Our results will show how useful this method can be in bridging the multidisciplinary gap of eHealth research and development, to inform and advance the importance of holistic approaches, while showcasing this approach for the case of self-management in cardiovascular diseases.

**Trial Registration:**

PROSPERO CRD42018104397; https://www.crd.york.ac.uk/PROSPERO/display_record.php? RecordID=104397 (Archived by WebCite at http://www.webcitation.org/75H1kP1Mm)

**International Registered Report Identifier (IRRID):**

DERR1-10.2196/13334

## Introduction

### Holistic Electronic Health Research and Development

Electronic health (eHealth) can be defined as the use of technology to support health, well-being, and health care [1]. As a field of science and innovation, eHealth is characterized by its multidisciplinary and rapidly evolving nature. In eHealth development, various disciplines such as computer, health, and behavioral sciences and design are involved. Ideally, researchers and practitioners are frequently engaged in iterative phases of eHealth development, implementation, or evaluation. The knowledge and technology generated by such processes is often grounded in a wide and overwhelming variety of frameworks, models, theories, methods, or guidelines. Because of this, accumulation, curation, and translation of the output of research and development has become a challenge and thus an important target for research itself [2].

Research has also made it clear that development of eHealth entails several challenges, such as maintaining the pace and efficiency of development cycles, promoting engagement, and applying a theoretical foundation [2]. In practical terms, multidisciplinary teams (health care providers, software developers, etc) are confronted with the need to determine the best approach for a project very early in the process. They are required to define the aims, the methods, and the overarching process that will guide development. Thus, frameworks, models, or theories not only facilitate the task, but also can increase the success of eHealth. Success in research and development can be determined by how much an intervention improves health and well-being (effectiveness), but also by providing explanations and advancing scientific knowledge on “what works for whom in what settings to change what behaviors, and how?” [2].

A holistic approach that combines multidisciplinary knowledge with novel methods and techniques is recommended to tackle the various development challenges and to ensure the effectiveness and efficacy of eHealth [3]. The term holistic refers to the importance of the whole and the interdependence of its parts [3]. In other words, when developing, implementing, or evaluating eHealth, fragmented analysis should be avoided, and each part, with its reciprocal influence on other parts, should be emphasized (eg, across contextual, technological, and human levels) [4]. The usefulness of taking a holistic approach was recently noted during the development of a framework to understand the nonadoption, abandonment, scale-up, spread, and sustainability of eHealth [5]. In the development process, a holistic view was a helpful starting point to analyze and understand data and theory, and to integrate other frameworks [5]. Therefore, we propose that both researchers and practitioners should recognize the value of making a conscious decision to strive for optimal holism, or at least to combine the most suitable, validated, and useful guidelines that reflect on their decision. Health care is a complex and adaptive system, and this makes eHealth a potential source for innovative solutions to some of society’s most alarming health care problems [6]. The Center for eHealth Research (CeHRes) roadmap is an example of a holistic approach built on reviews of previous frameworks and on empirical research that has been extensively employed for cases such as chronic diseases, antimicrobial stewardship programs [7], and others [3,4]. Thus, such a guideline offers researchers and developers several tools and methods to integrate into a project, in order to monitor the many different stakes and processes that are at play when tackling a certain health issue.

### Case Study: Self-Management of Cardiovascular Diseases Through Electronic Health Monitoring Technology

Cardiovascular diseases (CVDs) constitute a global health care crisis due to their high prevalence, long duration, and slow progression [8,9]. A key factor to lessen the burden of CVDs is to support the patients’ abilities to self-manage their own condition [10]. Self-management refers to an individual’s ability to manage the symptoms, treatment, and physical and psychosocial consequences, as well as the lifestyle changes inherent in living with a chronic condition [11]. For instance, individuals living with CVD are recommended to manage their blood pressure, control their cholesterol, reduce their blood sugar levels, become physically active, eat better, lose weight, and stop smoking [10]. An important aspect of these recommendations is that self-management has to be done outside the clinical setting, as patients have to integrate these intensive and timely activities into their daily lives. In fact, one estimate is that of the 8760 hours in a year, patients are spending only around 10 hours (0.1%) with their health care providers [10]. To ensure that patients are seen by or under the supervision of their health care providers when they do not have face-to-face contact, remote self-monitoring is crucial. Remote self-monitoring can be defined as the process of observing changes in signs and symptoms [12], a behavior that is primarily conducted by the patient but made visible to the health care providers via technology. It supports safety because the health care team can check and be alerted in a timely manner in case of potentially dangerous changes in the patient’s health status. Also, patients often feel more comfortable being able to return to their daily lives with the knowledge that important measurements are being monitored by their health care providers [13]. Because of this, remote self-monitoring technologies have become a vital part, almost a prerequisite, of home- and community-based care. In this light, recent metareviews have shown that technology-supported interventions can be at least as effective as usual care in supporting self-management of chronic conditions [14,15].

Despite promising results, the accumulation, curation, and translation of knowledge is also challenging when research in eHealth technology (computer science, design), CVDs (health sciences), and self-management (behavioral sciences) intersects. This leaves a gap that has been observed by previous reviews. The multidisciplinary gap is formed by the usage of different terms and concepts to explain the same phenomena [16], and by a lack of clarity or standardization in reporting the key ingredients of an intervention [17]. To exemplify from the behavioral science perspective, a review of eHealth physical activity interventions for adults with CVDs found that most studies did not sufficiently detail the operationalization of behavior change techniques as key components of their intervention [18]. Likewise, another review of similar interventions showed that only half of the studies had named a theory or model as the foundation [19].

The literature often provides lessons learned on a case-by-case basis in eHealth research and development to support self-management [14,15,20,21]. For example, the most common recommendations reflect the importance of applying technology integration models and a theoretical foundation. Even though this is valuable knowledge, testing should also include process evaluation for intermediate outcomes (mechanisms, mediators), derived ideally from the aforementioned theoretical background. Developers should also provide a sufficiently detailed description of the evidence-based components of the intervention (eg, behavior change techniques). Nevertheless, from these detached recommendations it is still unclear which overall development approaches have been applied in eHealth research to support self-management of CVD, and what their unique contributions have been. Even more so, the extent to which holistic principles have been considered is unknown. The uncertainty is highlighted because these interventions are coupled with rapidly evolving technologies such as body sensors, personalization algorithms, and automatic feedback systems [21] that mark a significant shift from the traditional telephone or face-to-face delivery. In sum, much is known about development processes in eHealth, based on the many examples that exist. What is lacking at this point is an overarching understanding that relates the findings of such studies across the phases of development and across disciplines.

### Aim and Focus

**Figure 1:**
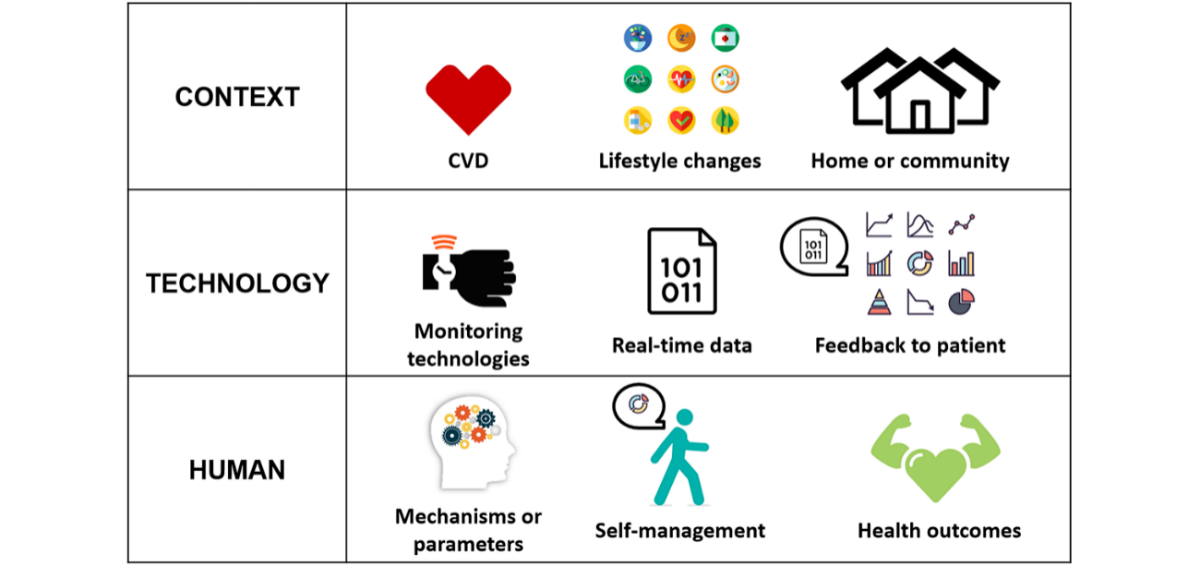
Holistic interpretation of electronic health monitoring technology to support self-management of cardiovascular disease (CVD).

The aim of this review is to facilitate a holistic interpretation of eHealth projects aimed at self-management support of CVDs in a natural setting of the patients. We intend to identify the frameworks, models, and theories applied in these projects and synthesize how their elements were applied to research and development. This seeks to fill in the gap of knowledge translation and dissemination resulting from the multidisciplinarity of eHealth. Figure 1 illustrates an initial framework of proposed interdependent elements for a holistic interpretation in terms of the context, the technology, and the human level.

As Figure 1 shows, the context of the review is broad. It includes patients with any particular CVD who are faced with lifestyle changes inherent to their disease and who have to cope with them predominantly at home or in their communities (not in a clinical setting). In terms of technology, we have narrowed the review aim down to the use of remote monitoring technologies such as blood pressure monitors, weigh scales, or wearables, which collect real-time data and provide feedback to the patient as a key component. This scope allows for the collection of specific knowledge on self-management support in the context of remote care. Although excluding interventions that did not use monitoring technology could be seen as a limitation, we hold that any of these applications could, and more importantly should, still be adapted to remote care; therefore, we expect our findings to showcase the missing potential. Finally, in terms of the human element, the aim is specific but also difficult to identify in published studies. The human element is represented by theory-based ingredients such as profiling or tailoring mechanisms and parameters of effectiveness to target patients’ behavior change with the intention to improve health.

The review is focused on the following research questions. First, what frameworks, models, or theories have been used to develop, implement, or evaluate eHealth interventions to support self-management of patients with CVDs outside the clinical setting? Second, how do these models address the 5 principles of a holistic eHealth research and development approach (as depicted by the CeHRes roadmap [3,4])? Third, what parameters of effectiveness, profiling mechanisms, and target outcomes are used in these models to address heterogeneity between patients with CVD?

### Selecting Metaethnography (Phase 1)

Study reports of how researchers and practitioners applied frameworks, models, and theories are the qualitative data of interest for this review, which is thus based on metaethnography, a qualitative synthesis approach developed by Noblit and Hare [22]. Metaethnography is an interpretive approach to qualitative evidence synthesis that seeks to generate a new understanding of a topic, while preserving the social and theoretical contexts in which findings emerge [23]. Noblit and Hare outlined metaethnography as a 7-stage process that compares and analyzes texts, creates new interpretations in the process, and by doing this strives to build a holistic interpretation [22]. In practice, it mainly involves open coding to identify emergent categories and then constant comparison of key metaphors across studies. Key metaphors can be phrases, ideas, concepts, perspectives, organizers, or themes revealed by a study [22].

Both the guidelines on choosing qualitative evidence synthesis methods by Booth et al [24] and the support of an information specialist for social sciences (PDN) led us to choose metaethnography over other approaches (eg, grounded theory or critical interpretive synthesis). We preferred metaethnography because it includes a synthesis approach matching the interest of the review to “move beyond description to a more interpretive examination of [themes,] their relationships and indeed any inherent contradictions” (pg 48) [23]. More importantly, metaethnography is by its very essence a technique used to translate concepts across individual studies [23], which is a perfect fit for our aim to synthesize the elements of frameworks, model, or theories. Our review is also informed by metaethnographies in related topics or with similar aims [25-29].

## Methods

**Figure 2:**
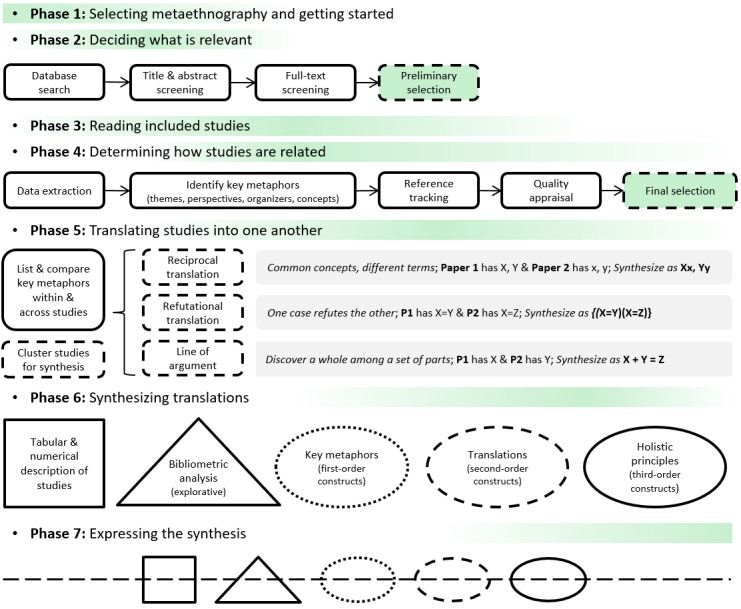
Metaethnography review of frameworks, models, and theories in electronic health research and development.

This protocol is in accordance with the recently developed Meta-ethnography Reporting Guidance (eMERGe) for metaethnographic studies [30]. Phase 1 (selecting metaethnography and getting started) is embodied in the Introduction; we describe the rest of the reporting criteria below. Figure 2 overviews the practical steps of the methodology.

### Search Strategy (Phase 2)

In phase 2, we will conduct an exhaustive search to find published studies of interest. The search will consist of (1) a systematic database literature search, followed by (2) backward and forward reference tracking from selected articles. The databases that we will search are Scopus, Web of Science, EMBASE, the CINAHL, PsycINFO, the ACM Digital Library, and the Cochrane Library. We chose Web of Science and Scopus based on their coverage of multidisciplinary fields of science, including technology, medicine, and social sciences. Both of these also cover MEDLINE, which is a database of interest due to its focus on the life sciences and biomedical literature. We selected EMBASE and CINAHL because of their discipline-specific literature on biomedicine and nursing, respectively. We included PsycINFO to ensure we would miss no studies from the behavioral field. Likewise, we included ACM Digital Library due to its focus on computer science. The Cochrane Library covers medicine and other health care specialties, including systematic reviews. We will adapt the search to the features of each database. In general, the main search limiters will be the time span (2008-2018) and the language (English, Dutch, or Spanish) of publication. We determined the time span of 10 years by taking into consideration the growth of the research field and the technological developments of interest. When possible, we will limit the search to articles that include an abstract and that are peer reviewed. The search will consist of multiple key terms. We chose terms based on the existing literature, as well as valuable synonyms of interest, and we will refine them through pilot searches. We will identify related terms and synonyms by using the Medical Subject Headings and EMBASE subject headings databases. The result is to be a very structured query consisting of 4 sets, aiming for results about frameworks, models, and theories (set 1), eHealth interventions (set 2), self-management (set 3), and CVDs (set 4).

We deem the probability of missing relevant articles after the systematic search, followed by the reference tracking and the screening procedure, to be negligible. We intend this strategy to identify articles and studies that add information about overarching eHealth projects within the scope of our review. We define a project as the overarching research project, usually identified by the name of the eHealth technology and integrating several research goals or development aims. The project can consist of 1 or more studies with specific aims (eg, usability or effectiveness). Finally, a study can be published in 1 or more articles (eg, protocol and results).

### Search Processes (Phase 2)

RRCM will search the databases and track the references. We will upload the database search results to EndNote X8 (Clarivate Analytics) and use the software features to eliminate duplicates.

### Selecting Studies (Phase 2)

We will select studies by uploading the citations to the Covidence Web-based software platform (Veritas Health Innovation Ltd). Articles will be screened by 2 reviewers, first by title and abstract, and then by full text. RRCM as the main reviewer will conduct the title and abstract screening stage throughout all the citations. RAA as the co-reviewer will screen 15% of the citations by default order of appearance in Covidence (alphabetically by first author’s name) and will discuss any discrepancies with RRCM to fine-tune the selection criteria. The selection criteria will ensure that the selected article fit within the interest of the review in terms of the population and context (eg, CVDs as a target group), the intervention (eg, self-management support through eHealth), the content of interest for the synthesis (eg, a framework, model, or theory applied and sufficiently described), and the study characteristics (eg, date and language of publication). Multimedia Appendix 1 lists the full inclusion and exclusion criteria. Covidence software allows for selecting articles on a “yes,” “no,” or “maybe” basis. Therefore, to validate the 85% of citations that will be screened by only the main reviewer, those tagged as “maybe” from the single review will also be screened by the co-reviewer. The full text of articles will be screened using the same approach. Discrepancies in article selection at all stages will be resolved in consultation with RS and LGP. We will present the outcome of the systematic search and selection process in the final report following the Preferred Reporting Items for Systematic Reviews and Meta-Analyses guidelines [31] (eg, flowchart), giving reasons for exclusion at full-text screening, especially for articles on which the reviewers did not reach agreement at once.

### Reading Studies and Extracting Data (Phases 3 and 4)

RRCM will conduct phases 3 and 4, and RAA, JW, RS, and LGP will provide feedback on the growing output at intervals. We will use a data extraction form based mainly on elements of the Consolidated Standards of Reporting Trials of Electronic and Mobile Health Applications and Online Telehealth (CONSORT-EHEALTH) checklist v.1.6 [32,33] and adapted to fit the aims of the review. We chose the CONSORT-EHEALTH checklist as the base because it is an accepted standard for reporting on eHealth studies. Because this standard was created for describing trials, we adapted it to reflect the aims of this review. Multimedia Appendix 2 shows the resultant data extraction form. To increase its validity, we will pilot test this form on a first sample of selected articles and iteratively adjust it as necessary during the data extraction process. We will record all changes and report them together with the results, in order to reflect on the usefulness of the extraction form. The form is designed to collect information about (1) the study description, (2) eHealth intervention, and (3) underlying framework, model, or theory, and (4) their principles and key elements according to a holistic perspective. The broadness of the data extraction form is intended to preserve the context of the research and development process as described in the selected article.

**Figure 3:**
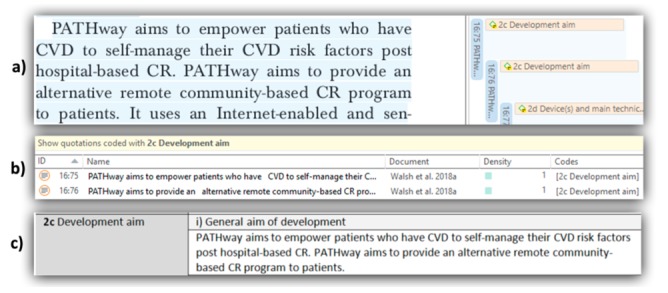
Example of the data extraction flow using ATLAS.ti and Microsoft Word. CR: cardiac rehabilitation; CVD: cardiovascular disease.

We will extract data using the qualitative software package ATLAS.ti version 8 (ATLAS.ti Scientific Software Development GmbH) and Microsoft Word 2016 (Microsoft Corporation). To begin, we will import PDF versions of the selected articles to ATLAS.ti and set up codes to reflect each element of the data extraction form. Figure 3 shows an example of how the data will flow through the data extraction approach. To facilitate a close and critical reading, this stage will consist of the following steps. First, the reviewer will read the article and code it at the same time according to the elements of the data extraction form (Figure 3, part a). We will also use open coding at this point for potential key concepts or ideas (metaphors). Second, after the article has been read and coded, we will use the quotation manager tool of ATLAS.ti to review the coding results per category (Figure 3, part b). For example, if nothing is coded for “General aim of development,” the reviewer will screen the article again to ascertain whether this element was skipped while reading or if it was not reported by the authors. This process will be repeated for every element of the data extraction form. In the third and final step, the reviewer will translate the coded data into a data extraction form in Microsoft Word (Figure 3, part c). This means that, for each selected article, there will be a data extraction form filled in with all the data of interest. The process will be iterative, as RRCM will continually cross-reference and refine the coding of the article and the data extraction form. RAA will independently revise the accuracy of this process by contrasting the first set of articles with each of their corresponding data extraction forms.

RRCM will also assess the quality of all selected articles using the Critical Appraisal Skills Programme’s checklists. We selected these checklists because they are a suggested and frequently used tool for metaethnographies [25,29,34-39]. Although many qualitative evidence synthesis studies do not appraise qualitative research [40], and while existing checklists often don’t match the goal of an individual qualitative evidence synthesis, it is considered good practice to apply it, even if in an adapted form. In addition, in the range of qualitative evidence synthesis methods, metaethnography is considered to have an objective idealism grounding (the acknowledgement that a world of collectively shared understandings exists) [38], which makes subjectivity more acceptable and puts relevance as the main inclusion criterion. Therefore, this step will not exclude any articles based on (methodological) quality, but we will keep it to encourage the reviewers to read the articles carefully and systematically [25]. In other words, articles at this stage will be considered a preselection, as they could still be excluded on the grounds of lack of relevance for the synthesis, which will be determined during the following phases (see Figure 2). We will present the characteristics of the selected articles for phase 3 in tabular and narrative format by year of publication; author(s); author’s affiliation(s) (institutions and countries); journal of publication; target condition; aim; and methodological design.

### Determining How Studies are Related (Phase 4)

Phase 4 will begin with the data extraction and thus overlaps with the reading of the studies. The main aspect of the studies to be compared will be the underlying framework, model, or theory applied, as well as the identified holistic principles and other key elements that influenced the eHealth development, implementation, or evaluation process. To make this process possible, the data extraction form is designed to identify such elements. In the data extraction form for each article, the reviewer will add notes when necessary to clarify annotations, for example, if the reviewer has to identify and screen the original source of a framework cited in the article to contrast how it is reported in the article (eg, to determine whether all elements of the framework are considered or only some of them).

**Figure 4:**
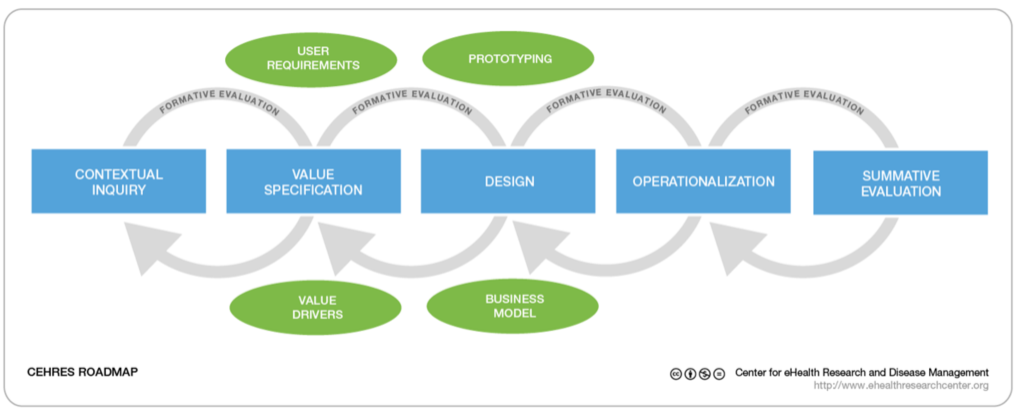
Center for eHealth Research (CeHRes) roadmap. Reproduced from van Gemert-Pijnen et al [4].

To facilitate the characterization of frameworks, models, and theories according to a holistic view, we will apply the principles of the CeHRes roadmap [3,4] as an initial interpretive framework. The roadmap is itself based on a review of multiple frameworks and was defined as the integration of persuasive technology design, human-centered design, and business modeling. It proposes 5 principles for eHealth development: (1) eHealth development is a participatory development process; (2) eHealth development creates new infrastructures for improving health care, health, and well-being; (3) eHealth development is intertwined with implementation; (4) eHealth development is coupled with persuasive design [41]; and (5) eHealth development requires continuous evaluation cycles. The principles of the roadmap underpin several stages and recommended activities for development (Figure 4).

For the purpose of the review, we are using the CeHRes roadmap as an initial lens that the reviewers will apply to relate the studies and to identify new, more case-specific principles, or even gaps in the literature. We will use a list of data extraction key terms and definitions to facilitate the characterization of the frameworks, models, and theories applied in the selected articles (Multimedia Appendix 3). The terms are grounded in the conceptualizations of the CeHRes roadmap [3,4] but are also informed by the multidisciplinary literature related to eHealth and intervention development [16,42,43].

To visualize and compare the data extracted per article and per project, we will use a matrix in Microsoft Excel 2016 (Multimedia Appendix 4). The matrix comparison will illustrate the reviewer’s characterization of the frameworks, models, and theories reported in the articles. Therefore, the matrix will allow for a first analysis of the clarity and extent of the data that can be synthesized. Multimedia Appendix 4 also shows a worked example of this. This visualization and the key metaphors that are open coded will be the basis to transition from the preselected pool of studies to the final selection of articles included in the synthesis.

The relation assessed between studies via the matrix will be complemented by 2 more tables. The first table will provide an overview of the eHealth projects by name of project; developers, sponsors, or owners; development aim; device(s) and main technical functionalities; main content feature(s) (eg, behavior change techniques); mode of delivery and implementation (eg, use parameters); and type of feedback (eg, blended care vs automated). The second table will present the frameworks, models, and theories identified by name; categorization (framework, theory, or model); studies and projects that applied it; approach to eHealth (development, implementation, or evaluation); coverage of CeHRes’s 5 holistic principles; and coverage of key elements to ensure effectiveness (behavior change, technology adoption, and outcomes). To complete this phase, JMN will conduct an explorative bibliometric analysis of the preselected pool of studies to accompany the study descriptions. This will be intended to identify the convergent points of the literature (eg, through a topic analysis or co-citation of journals) and identify potential biases or missing articles. This will also contribute to visualization of the context of the selected articles, especially the fields of science from which they draw knowledge and the common terms they share.

### Translating Studies (Phase 5)

RRCM will conduct phases 5 and 6, supported by RAA, JW, RS, and LGP in assessing and refining the output. We will attempt the translation process through various techniques known from the metaethnography literature [22,25-29,34-37,39,44-46], for instance, by the constant comparative method, making a list of key metaphors and comparing this across all studies. Alternatively, the translation process can be by choosing an index article and translating this to another study, then translating this first translation to a third study. If necessary, we will cluster articles to facilitate translation. For example, if several frameworks recommend a step of contextualization or a needs assessment with the target group, these elements could be translated to the principle of participatory development.

We will use concept maps or other forms of visual diagrams to describe the context and the meaning of the relationships between concepts within and across studies. We will consider potential alternative interpretations or explanations in the translation and present them in the final results.

### Synthesizing Translations (Phase 6)

During phase 6, we will compose the synthesis, as much as possible, in the language of holistic principles as depicted in the CeHRes roadmap. Therefore, we expect to conduct a line-of-argument synthesis (assuming that studies contribute to a shared line of thought) given the aim to provide a holistic view of the scope of the study. In any case, we will also apply a reciprocal and refutational analysis and add this to our general synthesis. For example, the synthesis could be structured according to the 5 principles of CeHRes and the content derived from the specific approaches of the selected projects. Similar to the previous step, potential alternative interpretations or explanations will be considered and presented.

We will present the new interpretation not as a newly developed metaframework, model, or theory, but rather as a set of principles synthesized from the literature about how to select, operationalize, and execute a holistic eHealth research and development process for the case of self-management of CVD in a natural setting. For example, the synthesis can provide information about commonly used methods through which business modeling can be integrated into a holistic development of an eHealth intervention to support patients with CVD at their home.

### Expressing the Synthesis (Phase 7)

We will submit our results to a peer-reviewed scientific journal that can potentially reach the multidisciplinary fields of science involved in eHealth (computer, health, and behavioral sciences, design, and others). We will contrast findings with the background literature to assess whether we have achieved a new interpretation or new knowledge. We will report the strengths and limitations of our review, and a general reflection on the metaethnography approach, focused on discussing its feasibility and usefulness for the field of eHealth. We will provide recommendations and conclusions based on the findings of the synthesis. These will include an overview of our future projects and how the metaethnographic synthesis might contribute to them.

## Results

We conceived the review early in 2018 and conducted the search in July (Multimedia Appendix 5 shows a complete and detailed list of the search terms we used, as well as the search strings for each database). By December 2018, we had completed phases 1 to 3; phase 4 is in its final stage. The database search yielded 1224 citations after we removed duplicates. After we applied the selection criteria, 17 articles remained. We have read and coded these articles, and are in the process of mapping them onto the data extraction matrix. We expect to submit the final results for publication in 2019.

## Discussion

This protocol describes a methodological adaptation of the metaethnography approach that serves the purpose of the review: a holistic interpretation of a multidisciplinary and rapidly evolving topic. This is why we conducted an exhaustive systematic search to find published studies within the scope. The main variation from other systematic reviews lies in the synthesis approach of metaethnography, which seeks to preserve the context in which findings have emerged from the various research disciplines at the crossroads of eHealth, CVDs, and self-management. In other words, the conceptual richness of the literature is needed to identify and understand the role of frameworks, models, and theories in the development of eHealth interventions. This wouldn’t be possible by aggregative methodologies or purely descriptive approaches. Furthermore, this review will show how several types of software (Covidence, ATLAS.ti, and Microsoft Office) can be employed to conduct as thorough a systematic qualitative evidence synthesis as metaethnography demands. Several steps not unique to metaethnography are also applied (quality appraisal, data extraction matrix, and bibliometric analysis) to provide clarity and depth to the analysis and synthesis. Finally, of added value is that the review adheres to the recently developed eMERGe [30] for metaethnographies. Our results will show how this method can contribute to overcoming the challenges derived from the multidisciplinary and rapidly evolving nature of eHealth research and development.

## Appendix

Multimedia Appendix 1 Inclusion and exclusion criteria.

Multimedia Appendix 2 Data extraction form.

Multimedia Appendix 3 Key terms and definitions for data extraction.

Multimedia Appendix 4 Template and worked example of the data extraction matrix.

Multimedia Appendix 5 Key and related terms, and full search strings per database.

## Abbreviations

CeHRes: Center for eHealth Research
CONSORT-EHEALTH: Consolidated Standards of Reporting Trials of Electronic and Mobile Health Applications and Online Telehealth
CVD: cardiovascular disease
eHealth: electronic health
eMERGe: Meta-ethnography Reporting Guidance
