# Supplementary material for: Frameworks, Models, and Theories Used in Electronic Health Research and Development to Support Self-Management of Cardiovascular Diseases Through Remote Monitoring Technologies: Protocol for a Metaethnography Review
Source: JMIR Res Protoc. 2019 Jul 16;8(7):e13334. doi: 10.2196/13334 (PMC6664658; doi:10.2196/13334)
Supplement: Multimedia Appendix 1 [file resprot_v8i7e13334_app1.docx]

## Multimedia Appendix 1 – Inclusion and exclusion criteria

### Instructions for Covidence

**Yes** = All criteria sufficiently met

**Maybe** = One criterion can't be clearly established (A note will be added by the reviewer)

**No** = Two or more criteria are not sufficiently met

* Criteria are hierarchically ordered, thus during full text review and in case of multiple exclusion reasons, please report it based on this sequence (e.g., 1a before 1c)

----------

**Population/Context**

1a. Focuses on cardiovascular diseases (Also when specifically mentioned: heart failure, hypertension, atrial fibrillation, coronary artery disease, peripheral artery disease; Include also if focused on CVD risk factors as long as the target is clear and specific)

1b. Focuses or includes target groups located outside the clinical setting (e.g., at home or in a community)

1c. Target group is treated mainly within a clinical setting (e.g., hospital inpatients)

**Intervention**

2a. Refers to an eHealth supported intervention that focuses or includes self-management support as a key component (Consider as equivalent terms: self-care, self-treatment, self-regulation, self-help, self-monitoring, self-medication; Include if mentioned in terms of the patient's perspective: disease management, disease controllability)

2b. The eHealth technology provides feedback to the patient based on self-monitoring data (collected via remote monitoring technologies such as wearables, blood pressure monitors, or weigh scale)

2c. Focuses only on disease management from the healthcare provider's (HCP) perspective

2d. Feedback is provided only via remote consultation with the HCP (e.g., by chat, telephone or video)

2e. Self-monitoring is performed only by self-reports and not by the use of a remote monitoring technology

**Content of interest**

3a. Refers to the use of a framework, model or theory applied to develop, implement, or evaluate the eHealth technology (Include when specifically mentioned: participatory design, persuasive design, user or human centred, and business modelling)

3b. The framework, model or theory is focused on healthcare service delivery (e.g., Chronic Care Model) or economic evaluation rather than on design or development guidelines for an eHealth supported intervention (e.g., participatory design, iterative evaluations)

3c. **[Full text screening only]** The framework, model or theory is not sufficiently described or not enough information is provided about its operationalization. **Sufficiency** is determined if two sub-criteria are met: **I)** The article includes a section that describes how a framework, model or theory was operationalized or applied for the development, implementation or evaluation of the intervention (e.g., a design/intervention section that describes development procedures, or a methods section that describes an –iterative– evaluation process). **II)** The article provides a description or background information about the underlying framework, model or theory applied (within the same text, via supplementary materials or by references to the original sources).

**Study characteristics**

4a. Quantitative or qualitative (Including protocols, reviews and articles published in conference proceedings) (Abstracts of conference proceedings are included if they hint towards an article of possible interest, which can be screened during the full text stage)

4b. It is published before 2008

4c. **[Full text screening only]** Full text cannot be accessed

4d. **[Full text screening only]** It is not written in English, Dutch or Spanish language

4e. **[Full text screening only]** It is a doctoral thesis

4f. **[Full text screening only]** It is not peer reviewed
