# Supplementary material for: Frameworks, Models, and Theories Used in Electronic Health Research and Development to Support Self-Management of Cardiovascular Diseases Through Remote Monitoring Technologies: Protocol for a Metaethnography Review
Source: JMIR Res Protoc. 2019 Jul 16;8(7):e13334. doi: 10.2196/13334 (PMC6664658; doi:10.2196/13334)
Supplement: Multimedia Appendix 2 [file resprot_v8i7e13334_app2.docx]

## Multimedia Appendix 2 – Data extraction form

The present data extraction form is mainly based on the CONSORT-EHEALTH checklist v.1.6., furtherly adapted to facilitate the aim of the present review. A relation between the research questions and the data extraction categories is presented below:

| **Research question** |  | **Data extraction category** |
| --- | --- | --- |
| - What frameworks, models, or theories have been used to develop, implement or evaluate eHealth interventions to support self-management of patients with CVD outside the clinical setting? | 🡺 | I. Study description  II. eHealth intervention  III. Underlying framework, model or theory |
| - How are the five principles of a holistic research and development approach of eHealth (as depicted by the CeHRes Roadmap) addressed in these models? | 🡺 | IV. Principles & key elements: **4a** |
| - What parameters of effectiveness, profiling mechanisms and target outcomes are used in these models to address heterogeneity between patients with CVD? | 🡺 | IV. Principles & key elements: **4b** |

The form was first piloted with a first sample of selected studies and also iteratively adjusted as necessary during the data extraction process.

| **I. Study description** | | |
| --- | --- | --- |
| **1a** Title |  | |
| **1b** Author(s) |  | |
| **1c** Affiliation(s) | i) Author(s) affiliations. *Include institutions and countries and mark the corresponding author.* | |
|  |  | |
|  | ii) Reported conflicts of interest | |
|  |  | |
| **1d** Year of publication |  | |
| **1e** Journal | i) Name |  |
|  | ii) Focus and scope. *Extracted from journal’s website* | |
|  |  | |
| **1f** Target condition(s) |  | |
| **1g** Aim | i) General study aim. *Including description of overarching or related project(s)* | |
|  |  | |
|  | ii) Research question(s) and study objective(s) | |
|  |  | |
| **1h** Design | i) Study classification. *Based on the Oxford Centre for Evidence-based medicine (*[*https://www.cebm.net/2014/04/study-designs/*](https://www.cebm.net/2014/04/study-designs/)*). If necessary, clarify if design is cross-sectional or longitudinal, prospective or retrospective, single or multi-group, randomized or non-randomized, and if blinded or open-label.* | |
|  |  | |
|  | ii) Setting. *General description of the organization of the study, including location(s) were study was conducted.* | |
|  |  | |
|  | iii) Institutions involved. *Hospitals, universities or other organizations involved. Which roles they had and how (if) these affiliations were displayed to participants.* | |
|  |  | |
| **1i** Participants | i) Eligibility criteria | |
|  |  | |
|  | ii) Recruitment procedure. *How participants were recruited (online vs. offline). If online-only, clarify if there were any anonymization measures. How participants were briefed for recruitment and in the informed consent procedures. Ethical approval information (if applicable).* | |
|  |  | |
|  | iii) Sample characteristics. *Baseline demographics, size, and other reported data.* | |
|  |  | |
|  | iv) Computer / Internet literacy | |
|  |  | |
| **1j** Study outcomes | i) Primary outcome(s) | |
|  |  | |
|  | ii) Secondary outcomes(s) | |
|  |  | |
|  | iii) Process outcome(s). *Including use or adoption metrics and how they were defined (e.g., what was considered a ‘session’)* | |
|  |  | |
|  | iv) Data collection method(s) and tools. *How outcomes were (self-)assessed, measured or monitored.* | |
|  |  | |

| **II. eHealth intervention** | |
| --- | --- |
| **2a** Name |  |
| **2b** Developers, sponsors, and owners | i) Developers & sponsors. *Clarify the relation of the study team towards the system being*  *evaluated. For example, if the authors are distinct from or identical with the developers of the intervention.* |
|  |  |
|  | ii) Owners. *Include names, credential and affiliations. Clarify if intervention/technology is still available and provide links for additional information if necessary.* |
|  |  |
| **2c** Development aim | i) General aim of development |
|  |  |
|  | ii) Specific objectives of development |
|  |  |
| **2d** Device(s) and main technical functionalities | *Include description (if) of interoperability between technological devices.* |
|  |  |
| **2e** Main content features | i) Summary of main content features |
|  |  |
|  | ii) In-depth description of content components. *Including behavior change techniques or persuasive design features with author(s) definitions. How (if) each component was tailored to individual circumstances.* |
|  |  |
| **2f** Mode of delivery and implementation | i) How participants accessed the intervention. *Required credentials to access the intervention components (e.g., web-based platform). Include if they had to pay (or were paid) to become members of a specific group.* |
|  |  |
|  | ii) Use parameters. *Intended ‘doses’ and optimal timing for use.* |
|  |  |
|  | iii) Instructions of use given to participants. *Such as timing, frequency or heaviness of use.* |
|  |  |
| **2g** Feedback | i) Main description of feedback process and features |
|  |  |
|  | ii) Level of human involvement. Automated only vs. blended care. *Number, specific roles and type of assistance of humans involved (e.g., care providers, health professionals, technical assistants), and medium by which involvement occurred.* |
|  |  |
|  | iii) Communication channels. *Synchronous vs asynchronous. Textual vs. visual. If prompts or reminders were used and what triggered them (e.g., frequency)* |
|  |  |
|  | iv) Presentation principles or strategies. *Descriptive information about the design and aesthetics of the intervention. Include principles or strategies used in page design, as well as basic information such as average amount of text on pages.* |
|  |  |
| **2h** Development process | i) Historical summary. *Narrative commentaries, notes, and observations from the authors about the development process.* |
|  |  |
|  | ii) Formative evaluations. *Include list of reported methods with keywords (e.g., focus groups, usability testing)* |
|  |  |
|  | iii) Digital preservation. *Include URL of the application, archived public materials (links to screenshots/videos/demo pages)* |
|  |  |
|  | iv) Published studies or grey literature. *List of related works (by authorship or project). Include and mark references that were also screened for inclusion in the present review.* |
|  |  |
| **2i** Intervention results | i) Results on primary and secondary outcome(s) |
|  |  |
|  | ii) Report on process outcome(s). *Including attrition.* |
|  |  |
|  | iii) Report on technical problems or unintended effects. *Not only including physical “harm” to participants, but also incidents such as perceived or real privacy breaches and other unexpected/unintended incidents.* |
|  |  |
|  | iv) Interpretation and principal findings. *Presented and summarized as per study question. Include limitations of study/project when reported, as well as unanswered new questions and suggestions for future research.* |
|  |  |

| **III. Underlying framework, model or theory** | | |
| --- | --- | --- |
| **3a** Name |  | |
| **3b** Description | i) Original source(s) referenced by the study author(s) | |
|  |  | |
|  | ii) General description. *If provided by selected study, otherwise cite original source.* | |
|  |  | |
|  | iii) Key framework, model or theory elements. *List of main principles, assumptions, concepts, components, parameters, conditions, phases or stages. Include definitions of each preferably provided by selected study, otherwise cite original source.* | |
|  |  | |
|  | iv) Visual representation. *If applicable and provided by selected study, otherwise cite original source.* | |
|  |  | |
| **3c** Operationalization | List author(s) statements regarding use or operationalization of the framework, model or theory. *Key elements listed in* ***3b-iii*** *are highlighted in bold.* | |
|  |  | |
| While **3a**, **3b**, and **3c** are strictly grounded in the content of the published study. **3d**, **3e**, **4a**, and **4b** are a preliminary framing by the reviewer to characterize the framework, model or theory in terms of its **application to eHealth**. | | |
| **3d** Categorization | Mark with an X if it meets any of the following definitions: | |
|  |  | Framework. *An* ***extensive*** s*et of principles, such as assumptions, constructs, quality criteria, and ideas that can guide research and development. It can also contain strategies such as guidelines, design heuristics, and methods to assist on a* ***staged****,* ***phased****, or* ***time*** ***oriented*** ***process****.* |
|  |  | Model. *A* ***simplified representation*** *of a reality, hypothesis, theory, or knowledge. It can contain a set of concepts, statements, or both that specify how constructs relate to each other. Although, it can be both* ***‘precise and quantified’*** *or ‘****imprecise and qualitative’****.* |
|  |  | Theory. *Set of concepts and/or statements with* ***specification of how phenomena relate to each other****. Theory provides an organizing description of a system that accounts for what is known, and* ***explains and predicts phenomena****.* |
| **3e** Approach to eHealth | Mark with an X if framework, model or theory was applied to any of the following: | |
|  |  | Development. *Refers to an* ***iterative process*** *of development of eHealth, entailing activities for pre-design, design, implementation and evaluation.* |
|  |  | Implementation. *Refers* ***exclusively*** *to activities that are undertaken to realize the adoption, dissemination and long-term use of a product in its intended context.* |
|  |  | Evaluation. *Refers* ***exclusively*** *to formative evaluation or summative evaluation.* ***Formative evaluation*** *englobes the activities throughout the entire development process that provide ongoing information on how to improve the development process, outcomes of activities and eHealth technology.* ***Summative evaluation*** *is the development phase which studies the influence and role of the technology on health, the context, behavior and stakeholder perspective via evaluations of impact and uptake of the technology.* |

| **IV. Principles & key elements** | | |
| --- | --- | --- |
| **4a** Key element(s) addressing CeHRes Roadmap principles | Mark with an X if a principle is met and name the key elements **(3b-iii)** that relate to it: | |
|  |  | Participatory development. *The structural cooperation of eHealth developers with potential end users and other stakeholders during its development; Including also user or human centred design, both generally defined as a framework that aims to develop solutions to problems by involving the human perspectives in all steps of the process, via observing the problem within context, brainstorming, conceptualizing, developing and implementing the solution.* |
|  | Elements |  |
|  |  | Persuasive technology design. *Designing technology that aims to reinforce, change, shape or influence behavior and attitudes by being compelling and without being coercive or deceptive.* |
|  | Elements |  |
|  |  | Business modelling. *Description of how an organization creates, delivers, and captures values. It can be a conceptual and analytical framework to discuss the added values of an eHealth intervention.* |
|  | Elements |  |
|  |  | Intertwined with implementation. *Inclusion in the development process of activities that are undertaken to realize the adoption, dissemination and long-term use of a product in its intended context.* |
|  | Elements |  |
|  |  | Continuous evaluation cycles. *Employment of iterative design methodologies based on a cyclic process of needs assessment, prototyping, testing, analyzing and refining a product, during which changes and refinements are made to the product based on the results of the most recent iteration of a design.* |
|  | Elements |  |
| **4b** Key elements addressed to ensure effectiveness of an intervention for CVD (and the heterogeneity of patients) | Behavior change. *List elements that refer to practical applications or parameters of effectiveness of behavior change methods as defined by Peters, de Bruin and Crutzen (2015, p.3): “****Practical applications*** *are the translations of theoretical methods of behavior change to practical intervention elements.* ***Applications*** *are by definition specific, ideally tailored to populations, intervention contexts and behavioral domains.* ***Parameters for effectiveness*** *are the characteristics that a practical application must manifest for it to accurately reflect the theoretical method. When these parameters are lost in translation from method to application, effective behavior change is undermined and may even result in counterproductive effects. Evidence for the existence of such parameters can range from theoretical to meta-analytical”.* | |
|  |  | |
|  | Technology adoption. *List elements that aim to increase the engagement, use, adherence, uptake or adoption of the technology. For example, the use of profiling mechanisms, defined as elements that are employed to adapt an eHealth intervention to the characteristics of an individual or cohort (e.g., motivation levels as measured in a pre-test).* | |
|  |  | |
|  | Outcomes. *List target outcomes of an intervention that directly or indirectly have an impact on the health or wellbeing of the target group. For example, changes in health parameters (e.g., blood pressure control), risk factors (e.g., weight), or performance of self-care or healthy behaviors (e.g., physical activity levels).* | |
|  |  | |
