# Supplementary material for: Frameworks, Models, and Theories Used in Electronic Health Research and Development to Support Self-Management of Cardiovascular Diseases Through Remote Monitoring Technologies: Protocol for a Metaethnography Review
Source: JMIR Res Protoc. 2019 Jul 16;8(7):e13334. doi: 10.2196/13334 (PMC6664658; doi:10.2196/13334)
Supplement: Multimedia Appendix 3 [file resprot_v8i7e13334_app3.docx]

## Multimedia Appendix 3 – Key terms and definitions for data extraction

### Categorization

A **framework** is defined as an *extensive* set of principles, such as assumptions, constructs, quality criteria, and ideas that can guide research and development. It can also contain strategies such as guidelines, design heuristics, and methods to assist on a *staged*, *phased*, or *time oriented process*.

A **model** is defined as a *simplified representation* of a reality, hypothesis, theory, or knowledge. It can contain a set of concepts, statements, or both that specify how constructs relate to each other. Although, it can be both *‘precise and quantified’* or *‘imprecise and qualitative’*.

**Theory** is defined as a set of concepts and/or statements with *specification of how phenomena relate to each other.* Theory provides an organizing description of a system that accounts for what is known, and *explains and predicts phenomena.*

### Approach to eHealth

**Development** refers to an *iterative process* of development of eHealth, entailing activities for pre-design, design, implementation and evaluation.

**Implementation** refers *exclusively* to activities that are undertaken to realize the adoption, dissemination and long-term use of a product in its intended context.

**Evaluation** refers *exclusively* to formative evaluation or summative evaluation. *Formative evaluation* englobes the activities throughout the entire development process that provide ongoing information on how to improve the development process, outcomes of activities and eHealth technology. *Summative evaluation* is the development phase which studies the influence and role of the technology on health, the context, behavior and stakeholder perspective via evaluations of impact and uptake of the technology.

### Holistic principles

**Participatory development** is defined as a structural cooperation of eHealth developers with potential end users and other stakeholders during its development.

**Human or user-centred design** is defined as a framework that aims to develop solutions to problems by involving the human perspectives in all steps of the process, via observing the problem within context, brainstorming, conceptualizing, developing and implementing the solution.

**Business modelling** is defined as how an organization creates, delivers and captures values. It can be a conceptual and analytical framework to discuss the added values of an eHealth intervention.

The principle of **persuasive technology design** refers to designing technology that aims to reinforce, change, shape or influence behavior and attitudes by being compelling and without being coercive or deceptive.

The principle of development being **intertwined with implementation** refers to the inclusion in the development process of activities that are undertaken to realize the adoption, dissemination and long-term use of a product in its intended context.

The principle of **continuous evaluation cycles** refers to the employment of iterative design methodologies based on a cyclic process of needs assessment, prototyping, testing, analyzing and refining a product, during which changes and refinements are made to the product based on the results of the most recent iteration of a design.

### Key elements addressed to ensure effectiveness of an intervention

**‘Behavior change’ key elements** refer to *practical applications* or *parameters of effectiveness of behavior change methods* as defined by Peters, de Bruin and Crutzen (2015, p.3): “**Practical applications** are the translations of theoretical methods of behavior change to practical intervention elements. Applications are by definition specific, ideally tailored to populations, intervention contexts and behavioral domains. **Parameters for effectiveness** are the characteristics that a practical application must manifest for it to accurately reflect the theoretical method. When these parameters are lost in translation from method to application, effective behavior change is undermined and may even result in counterproductive effects. Evidence for the existence of such parameters can range from theoretical to meta-analytical”.

**‘Technology adoption’ key elements** refer to those that aim to increase the engagement, use, adherence, uptake or adoption of the technology. For example, the use of profiling mechanisms, defined as elements that are employed to adapt an eHealth intervention to the characteristics of an individual or cohort (e.g., motivation levels as measured in a pre-test).

**‘Outcomes’ key elements** refer to the target outcomes of an intervention that directly or indirectly have an impact on the health or wellbeing of the target group. For example, changes in health parameters (e.g., blood pressure control), risk factors (e.g., weight), or performance of self-care or healthy behaviors (e.g., physical activity levels).
