# Supplementary material for: Frameworks, Models, and Theories Used in Electronic Health Research and Development to Support Self-Management of Cardiovascular Diseases Through Remote Monitoring Technologies: Protocol for a Metaethnography Review
Source: JMIR Res Protoc. 2019 Jul 16;8(7):e13334. doi: 10.2196/13334 (PMC6664658; doi:10.2196/13334)
Supplement: Multimedia Appendix 4 [file resprot_v8i7e13334_app4.docx]

## Multimedia Appendix 4 – Template and worked example of the data extraction matrix

In the matrix each row represents category and field of the data extraction and each column represents a single paper. The columns can be organized to cluster the selected papers that belong to a similar project. Each cell can be coded as follows: ‘Clear’ (Green) if the data element is clearly identifiable in the paper (e.g., the eligibility criteria of a sample of patients is reported); ‘Unclear’ (Yellow) if the data element is partially identifiable or incomplete in the paper (e.g., the characteristics of the sample are reported only scarcely and not with a detailed table or description in the text); ‘Not applicable or not reported’ (Red) if the data element does not apply or is not reported (e.g., the devices and main technical functionalities of the eHealth technology are not mentioned).

|  | **Paper** | *P1* | *P2* | *P3* | *P4* | *P5* |
| --- | --- | --- | --- | --- | --- | --- |
| **I. Study description** | **1a Title** |  |  |  |  |  |
|  | **1b Author(s)** |  |  |  |  |  |
| **1c Affiliation(s)** | i) Author(s) affiliations |  |  |  |  |  |
|  | ii) Reported conflicts of interest |  |  |  |  |  |
|  | 1d Year of publication |  |  |  |  |  |
| **1e Journal** | i) Name |  |  |  |  |  |
|  | ii) Focus and scope |  |  |  |  |  |
|  | **1f Target condition(s)** |  |  |  |  |  |
| **1g Aim** | i) General study aim |  |  |  |  |  |
|  | ii) Research question(s) and study objective(s) |  |  |  |  |  |
| **1h Design** | i) Study classification |  |  |  |  |  |
|  | ii) Setting |  |  |  |  |  |
|  | iii) Institutions involved |  |  |  |  |  |
| **1i Participants** | i) Eligibility criteria |  |  |  |  |  |
|  | ii) Recruitment procedure |  |  |  |  |  |
|  | iii) Sample characteristics |  |  |  |  |  |
|  | iv) Computer / Internet literacy |  |  |  |  |  |
| **1j Study outcomes** | i) Primary outcome(s) |  |  |  |  |  |
|  | ii) Secondary outcomes(s) |  |  |  |  |  |
|  | iii) Process outcome(s) |  |  |  |  |  |
|  | iv) Data collection method(s) and tools |  |  |  |  |  |
| **II. eHealth intervention** | **2a Name** |  |  |  |  |  |
| **2b Developers, sponsors, and owners** | i) Developers & sponsors |  |  |  |  |  |
|  | ii) Owners |  |  |  |  |  |
| **2c Development aim** | i) General aim of development |  |  |  |  |  |
|  | ii) Specific objectives of development |  |  |  |  |  |
|  | **2d Device(s) and main technical functionalities** |  |  |  |  |  |
| **2e Main content features** | i) Summary of main content features |  |  |  |  |  |
|  | ii) In-depth description of content components |  |  |  |  |  |
| **2f Mode of delivery and implementation** | i) How participants accessed the intervention |  |  |  |  |  |
|  | ii) Use parameters |  |  |  |  |  |
|  | iii) Instructions of use given to participants |  |  |  |  |  |
| **2g Feedback** | i) Main description of feedback process and features |  |  |  |  |  |
|  | ii) Level of human involvement |  |  |  |  |  |
|  | iii) Communication channels |  |  |  |  |  |
|  | iv) Presentation principles or strategies |  |  |  |  |  |
| **2h Development process** | i) Historical summary |  |  |  |  |  |
|  | ii) Formative evaluations |  |  |  |  |  |
|  | iii) Digital preservation |  |  |  |  |  |
|  | iv) Published studies or grey literature |  |  |  |  |  |
| **2i Intervention results** | i) Results on primary and secondary outcome(s) |  |  |  |  |  |
|  | ii) Report on process outcome(s). Including attrition |  |  |  |  |  |
|  | iii) Report on technical problems or unintended effects |  |  |  |  |  |
|  | iv) Interpretation and principal findings |  |  |  |  |  |
| **III. Underlying framework, model or theory** | **3a Name** |  |  |  |  |  |
| **3b Description** | i) Original source(s) referenced by the study author(s) |  |  |  |  |  |
|  | ii) General description |  |  |  |  |  |
|  | iii) Key framework, model or theory elements |  |  |  |  |  |
|  | iv) Visual representation |  |  |  |  |  |
|  | **3c Operationalization** |  |  |  |  |  |
| **3d Categorization** | Framework |  |  |  |  |  |
|  | Model |  |  |  |  |  |
|  | Theory |  |  |  |  |  |
| **3e Application for eHealth** | Development |  |  |  |  |  |
|  | Implementation |  |  |  |  |  |
|  | Evaluation |  |  |  |  |  |
| **IV. Principles & key elements** | Participatory development |  |  |  |  |  |
| **4a Key element(s) CeHRes principles** | Persuasive technology design |  |  |  |  |  |
|  | Business modelling |  |  |  |  |  |
|  | Intertwined with implementation |  |  |  |  |  |
|  | Continuous evaluation cycles |  |  |  |  |  |
| **4b Key elements to ensure effectiveness** | Behavior change |  |  |  |  |  |
|  | Technology adoption |  |  |  |  |  |
|  | Outcomes |  |  |  |  |  |

|  | **Project** | **Project A** | | | **Project B** | | |
| --- | --- | --- | --- | --- | --- | --- | --- |
|  | **Paper** | *P1* | *P2* | *P3* | *P4* | *P5* | *P6* |
| **I. Study description** | | | | | | | |
| 1d Year of publication | | **2016** | **2018** | **2018** | **2016** | **2015** | **2015** |
| **1f Target condition(s)** | | **HF** | **HF** | **HF** | **HF** | **HF** | **HF** |
| **1g Aim** | i) General study aim | A | A | A | A | A | A |
|  | ii) Research question(s) and study objective(s) | A | B | A | A | A | a |
| **1h Design** | ii) Setting | A | C | A | A | A | C |
|  | iii) Institutions involved | C | C | A | B | B | C |
| **1i Participants** | i) Eligibility criteria | C | C | B | A | A | A |
|  | ii) Recruitment procedure | A | C | A | A | A | A |
|  | iii) Sample characteristics | A | B | B | A | A | B |
|  | iv) Computer / Internet literacy | A | C | C | A | A | C |
| **1j Study outcomes** | i) Primary outcome(s) | A | B | B | A | A | A |
|  | ii) Secondary outcomes(s) | C | B | C | C | A | C |
|  | iii) Process outcome(s) | C | B | C | C | A | C |
|  | iv) Data collection method(s) and tools | A | B | A | A | A | A |
| **II. eHealth intervention** | | | | | | | |
| **2b Developers, sponsors, and owners** | i) Developers & sponsors | A | a | A | A | A | A |
|  | ii) Owners | B | B | B | C | C | C |
| **2c Development aim** | i) General aim of development | A | A | A | A | A | A |
|  | ii) Specific objectives of development | B | A | B | B | C | A |
| **2d Device(s) and main technical functionalities** | | A | A | C | A | A | A |
| **2e Main content features** | i) Summary of main content features | A | A | C | A | a | A |
|  | ii) In-depth description of content components | A | A | C | B | C | A |
| **2f Mode of delivery and implementation** | i) How participants accessed the intervention | B | C | C | C | B | A |
|  | ii) Use parameters | C | A | C | A | A | C |
|  | iii) Instructions of use given to participants | B | C | C | A | A | C |
| **2g Feedback** | i) Main description of feedback process and features | A | A | C | B | B | B |
|  | ii) Level of human involvement | B | C | C | B | A | B |
|  | iii) Communication channels | B | B | C | B | B | C |
|  | iv) Presentation principles or strategies | C | C | C | C | C | C |
| **2h Development process** | i) Historical summary | C | A | A | B | B | B |
|  | ii) Formative evaluations | B | A | B | A | A | A |
|  | iii) Digital preservation | C | C | C | B | C | C |
|  | iv) Published studies or grey literature | A | A | A | A | A | A |
| **2i Intervention results** | i) Results on primary and secondary outcome(s) | A | C | A | A | A | A |
|  | ii) Report on process outcome(s). Including attrition | C | C | C | C | A | C |
|  | iii) Report on technical problems or unintended effects | C | C | C | A | A | A |
|  | iv) Interpretation and principal findings | A | B | A | A | A | A |
| **III. Underlying frameworks, models or theories** | | | | | | | |
| **3b Description** | i) Original source(s) referenced by the study author(s) | A | A | B | A | A | A |
|  | ii) General description | A | A | B | A | B | A |
|  | iii) Key framework, model or theory elements | b | A | B | A | B | A |
|  | iv) Visual representation | b | A | A | B | B | A |
| **3c Operationalization** | | b | A | B | A | C | A |
| **3d Categorization** | Framework | **1** | **2** | **C** | **5** | **3** | **3** |
|  | Model | **C** | **C** | **1** | **C** | **1** | **C** |
|  | Theory | **1** | **1** | **C** | **1** | **C** | **C** |
| **3e Application for eHealth** | Development | **2** | **3** | **1** | **3** | **3** | **3** |
|  | Implementation | **C** | **1** | **C** | **1** | **3** | **3** |
|  | Evaluation | **C** | **1** | **C** | **2** | **3** | **3** |
| **IV. Principles & key elements** | | | | | | | |
| **4a Key element(s) CeHRes principles** | Participatory development | **C** | **1** | **C** | **5** | **3** | **3** |
|  | Persuasive technology design | **C** | **C** | **C** | **C** | **C** | **C** |
|  | Business modelling | **C** | **C** | **B** | **C** | **C** | **C** |
|  | Intertwined with implementation | **C** | **1** | **C** | **5** | **3** | **3** |
|  | Continuous evaluation cycles | **C** | **1** | **C** | **5** | **3** | **3** |
| **4b Key elements to ensure effectiveness** | Behavior change | **2** | **2** | **C** | **C** | **C** | **C** |
|  | Technology adoption | **C** | **C** | **C** | **1** | **1** | **C** |
|  | Outcomes | **2** | **2** | **C** | **C** | **C** | **C** |
| **Abbreviations:** HF = Heart failure. **Codes:** Clear Data element is clearly identifiable**;** Unclear Data element is partially identifiable or incomplete**;** Not applicable or missing data Data element does not apply or is not reported. **Numbers:** In section III, numbers quantify frameworks, models, or theories identified per paper (e.g., P1 includes data about one framework and one theory, both applied to development). In section IV, numbers quantify how many frameworks, models, or theories include certain key elements (e.g., P1 includes frameworks, models, or theories that include key elements addressing behavior change and outcome effectiveness). Therefore, numbers shouldn’t be read or added row-wise. For instance, because the framework identified in P1 is also identified in P2. | | | | | | | |
