# Supplementary material for: Frameworks, Models, and Theories Used in Electronic Health Research and Development to Support Self-Management of Cardiovascular Diseases Through Remote Monitoring Technologies: Protocol for a Metaethnography Review
Source: JMIR Res Protoc. 2019 Jul 16;8(7):e13334. doi: 10.2196/13334 (PMC6664658; doi:10.2196/13334)
Supplement: Multimedia Appendix 5 [file resprot_v8i7e13334_app5.docx]

## Multimedia Appendix 5 – Key and related terms and full search strings per database

| **Set 1** Key terms |
| --- |
| - Framework - Theory - Model - Participatory - Persuasive design - User centred - Human centred - Business modelling |

| **Set 2** Key term **^a^** and related terms | | | |
| --- | --- | --- | --- |
| - **eHealth ^a^** - e-health - electronic health - mHealth - m-health - mobile health - mobile application - mobile intervention - telehealth - telemedicine - telecare - telemonitoring - health informatics - medical informatics - informatics intervention - health care informatics - personal informatics - nursing informatics | - health enabling technology - health interactive technology - health information technology - health technology - assistive health technology - persuasive technology - persuasive health technology - technology-supported health - behavioral intervention technology - digital behavior change intervention - digital intervention | - behavior change support system - health care information system - interactive health system - decision support system - online system - computer system - communication system - information system - interactive health communication application - computer application - computer mediated communication | - therapy computer assisted - computer assisted - telecommunications media - health record - online service - online therapy - internet - web-based - website |

| **Set 3** Key term **^a^** and related terms |
| --- |
| - **self-management** **^a^** - self-care - self-treatment - self-regulation - self-help - self-monitoring - self-medication - disease management - disease controllability |

| **Set 4** Key term and related terms |
| --- |
| - **cardiovascular disease** **^a^** - cardiovascular disorder - heart disease - heart failure - cardiac failure - hypertension - atrial fibrillation - coronary artery disease - peripheral artery disease |

### Scopus: Advanced search

( TITLE-ABS-KEY ( fram* OR theor* OR model* OR "participatory" OR "persuasive" OR "user-cent*" OR "human-cent*" OR "business model*" ) ) AND ( TITLE-ABS-KEY ( ehealth* OR "e-health*" OR "electronic health*" OR mhealth* OR "m-health*" OR "mobil* health*" OR "mobil* application*" OR "mobil* intervention*" OR telehealth* OR "tele-health*" OR telemedic* OR "tele-medic*" OR telecar* OR "tele-car*" OR telemonitor* OR "tele-monitor*" OR "health informatic*" OR "medic* informatic*" OR "informatic* intervention*" OR "*care informatic*" OR "personal* informatic*" OR "nurs* informatic*" OR "enabl* technolog*" OR "health interact* technolog*" OR "health informat* technolog*" OR "health* technolog*" OR "assist* health* technolog*" OR "health* assist* technolog*" OR "persuas* technolog*" OR "persuas* health* technolog*" OR "technology-supported" OR "behavio* intervention* technolog*" OR "digital* behavio* change intervention*" OR "digital* intervention*" OR "behavio* change support system*" OR "*care information* system*" OR "interact* health* system*" OR "decision* support* system*" OR "online system*" OR "comput* system*" OR "communication system*" OR "information* system*" OR "interact* health* communication applicatio*" OR "comput* application*" OR "comput* mediat* communication*" OR "therap* comput* assist*" OR "comput* assist*" OR "telecommunication* media" OR "health* record*" OR "online service*" OR "online therap*" OR internet OR "web-based" OR website ) ) AND ( TITLE-ABS-KEY ( selfmanag* OR "self-manag*" OR selfcar* OR "self-car*" OR selftreat* OR "self-treat*" OR selfregulat* OR "self-regulat*" OR selfhelp* OR "self-help*" OR selfmonitor* OR "self-monitor*" OR selfmedicat* OR "self-medicat*" OR "disease manag*" OR "disease controllability" ) ) AND ( TITLE-ABS-KEY ( "cardi* disease*" OR "cardi* disorder*" OR "heart disease*" OR "heart failure" OR "cardi* failure" OR “hypertension” OR “atrial fibrillation” OR ”coronary artery disease” OR “peripheral artery disease” ) ) AND PUBYEAR > 2007

### Web of Science: All Databases, Timespan: 2008-2018

TS=(fram* OR theor* OR model* OR "participatory" OR "persuasive" OR "user-cent*" OR "human-cent*" OR "business model*") AND TS=(ehealth* OR "e-health*" OR "electronic health*" OR mhealth* OR "m-health*" OR "mobil* health*" OR "mobil* application*" OR "mobil* intervention*" OR telehealth* OR "tele-health*" OR telemedic* OR "tele-medic*" OR telecar* OR "tele-car*" OR telemonitor* OR "tele-monitor*" OR "health informatic*" OR "medic* informatic*" OR "informatic* intervention*" OR "*care informatic*" OR "personal* informatic*" OR "nurs* informatic*" OR "enabl* technolog*" OR "health interact* technolog*" OR "health informat* technolog*" OR "health* technolog*" OR "assist* health* technolog*" OR "health* assist* technolog*" OR "persuas* technolog*" OR "persuas* health* technolog*" OR "technology-supported" OR "behavio* intervention* technolog*" OR "digital* behavio* change intervention*" OR "digital* intervention*" OR "behavio* change support system*" OR "*care information* system*" OR "interact* health* system*" OR "decision* support* system*" OR "online system*" OR "comput* system*" OR "communication system*" OR "information* system*" OR "interact* health* communication applicatio*" OR "comput* application*" OR "comput* mediat* communication*" OR "therap* comput* assist*" OR "comput* assist*" OR "telecommunication* media" OR "health* record*" OR "online service*" OR "online therap*" OR internet OR "web-based" OR website) AND TS=(selfmanag* OR "self-manag*" OR selfcar* OR "self-car*" OR selftreat* OR "self-treat*" OR selfregulat* OR "self-regulat*" OR selfhelp* OR "self-help*" OR selfmonitor* OR "self-monitor*" OR selfmedicat* OR "self-medicat*" OR "disease manag*" OR "disease controllability") AND TS=("cardi* disease*" OR "cardi* disorder*" OR "heart disease*" OR "heart failure" OR "cardi* failure" OR “hypertension” OR “atrial fibrillation” OR ”coronary artery disease” OR “peripheral artery disease”)

### Embase: Advanced search

1. ('fram*' OR 'theor*' OR 'model*' OR 'participatory' OR 'persuasive' OR 'user-cent*' OR 'human-cent*' OR 'business model*':ab,kw,ti,cl,de) AND ([dutch]/lim OR [english]/lim OR [spanish]/lim)

2. (ehealth* OR 'e-health*' OR 'electronic health*' OR mhealth* OR 'm-health*' OR 'mobil* health*' OR 'mobil* application*' OR 'mobil* intervention*' OR telehealth* OR 'tele-health*' OR telemedic* OR 'tele-medic*' OR telecar* OR 'tele-car*' OR telemonitor* OR 'tele-monitor*' OR 'health informatic*' OR 'medic* informatic*' OR 'informatic* intervention*' OR 'healthcare informatic*' OR 'health-care informatic*' OR 'care informatic*' OR 'personal* informatic*' OR 'nurs* informatic*':ab,kw,ti,cl,de) AND ([dutch]/lim OR [english]/lim OR [spanish]/lim)

3. ('enabl* technolog*' OR 'health interact* technolog*' OR 'health informat* technolog*' OR 'health* technolog*' OR 'assist* health* technolog*' OR 'health* assist* technolog*' OR 'persuas* technolog*' OR 'persuas* health* technolog*' OR 'technology-supported' OR 'behavio* intervention* technolog*' OR 'digital* behavio* change intervention*' OR 'digital* intervention*' OR 'behavio* change support system*' OR 'healthcare information* system*' OR 'health-care information system*' OR 'care information system*' OR 'interact* health* system*' OR 'decision* support* system*' OR 'online system*' OR 'comput* system*' OR 'communication system*' OR 'information* system*':ab,kw,ti,cl,de) AND ([dutch]/lim OR [english]/lim OR [spanish]/lim)

4. ('interact* health* communication applicatio*' OR 'comput* application*' OR 'comput* mediat* communication*' OR 'therap* comput* assist*' OR 'comput* assist*' OR 'telecommunication* media' OR 'health* record*' OR 'online service*' OR 'online therap*' OR internet OR 'web-based' OR website:ab,kw,ti,cl,de) AND ([dutch]/lim OR [english]/lim OR [spanish]/lim)

5. #2 OR #3 OR #4

6. (selfmanag* OR 'self-manag*' OR selfcar* OR 'self-car*' OR selftreat* OR 'self-treat*' OR selfregulat* OR 'self-regulat*' OR selfhelp* OR 'self-help*' OR selfmonitor* OR 'self-monitor*' OR selfmedicat* OR 'self-medicat*' OR 'disease manag*' OR 'disease controllability':ab,kw,ti,cl,de) AND ([dutch]/lim OR [english]/lim OR [spanish]/lim)

7. ('cardi* disease*' OR 'cardi* disorder*' OR 'heart disease*' OR 'heart failure' OR 'cardi* failure' OR 'hypertension' OR 'atrial fibrillation' OR 'coronary artery disease' OR 'peripheral vascular disease':ab,kw,ti,cl,de) AND ([dutch]/lim OR [english]/lim OR [spanish]/lim)

8. #1 AND #5 AND #6 AND #7 AND [2008-2019]/py

### CINAHL (EbscoHost): Each set searched in title [TI] OR abstract [AB] OR subject [SU] and limited to ‘abstract available’, ‘peer reviewed’, and published at least from January 2008

1. fram* OR theor* OR model* OR "participatory" OR "persuasive" OR "user-cent*" OR "human-cent*" OR "business model*"

2. ehealth* OR "e-health*" OR "electronic health*" OR mhealth* OR "m-health*" OR "mobil* health*" OR "mobil* application*" OR "mobil* intervention*" OR telehealth* OR "tele-health*" OR telemedic* OR "tele-medic*" OR telecar* OR "tele-car*" OR telemonitor* OR "tele-monitor*" OR "health informatic*" OR "medic* informatic*" OR "informatic* intervention*" OR "*care informatic*" OR "personal* informatic*" OR "nurs* informatic*" OR "enabl* technolog*" OR "health interact* technolog*" OR "health informat* technolog*" OR "health* technolog*" OR "assist* health* technolog*" OR "health* assist* technolog*" OR "persuas* technolog*" OR "persuas* health* technolog*" OR "technology-supported" OR "behavio* intervention* technolog*" OR "digital* behavio* change intervention*" OR "digital* intervention*" OR "behavio* change support system*" OR "*care information* system*" OR "interact* health* system*" OR "decision* support* system*" OR "online system*" OR "comput* system*" OR "communication system*" OR "information* system*" OR "interact* health* communication applicatio*" OR "comput* application*" OR "comput* mediat* communication*" OR "therap* comput* assist*" OR "comput* assist*" OR "telecommunication* media" OR "health* record*" OR "online service*" OR "online therap*" OR internet OR "web-based" OR website

3. selfmanag* OR "self-manag*" OR selfcar* OR "self-car*" OR selftreat* OR "self-treat*" OR selfregulat* OR "self-regulat*" OR selfhelp* OR "self-help*" OR selfmonitor* OR "self-monitor*" OR selfmedicat* OR "self-medicat*" OR "disease manag*" OR "disease controllability"

4. "cardi* disease*" OR "cardi* disorder*" OR "heart disease*" OR "heart failure" OR "cardi* failure" OR “hypertension” OR “atrial fibrillation” OR ”coronary artery disease” OR “peripheral artery disease”

5. #1 AND #2 AND #3 AND #4

### PsycINFO (EbscoHost): Each set searched in title [TI] OR abstract [AB] OR keywords [KW] and limited to ‘peer reviewed’, and published at least from January 2008

1. fram* OR theor* OR model* OR "participatory" OR "persuasive" OR "user-cent*" OR "human-cent*" OR "business model*"

2. ehealth* OR "e-health*" OR "electronic health*" OR mhealth* OR "m-health*" OR "mobil* health*" OR "mobil* application*" OR "mobil* intervention*" OR telehealth* OR "tele-health*" OR telemedic* OR "tele-medic*" OR telecar* OR "tele-car*" OR telemonitor* OR "tele-monitor*" OR "health informatic*" OR "medic* informatic*" OR "informatic* intervention*" OR "*care informatic*" OR "personal* informatic*" OR "nurs* informatic*" OR "enabl* technolog*" OR "health interact* technolog*" OR "health informat* technolog*" OR "health* technolog*" OR "assist* health* technolog*" OR "health* assist* technolog*" OR "persuas* technolog*" OR "persuas* health* technolog*" OR "technology-supported" OR "behavio* intervention* technolog*" OR "digital* behavio* change intervention*" OR "digital* intervention*" OR "behavio* change support system*" OR "*care information* system*" OR "interact* health* system*" OR "decision* support* system*" OR "online system*" OR "comput* system*" OR "communication system*" OR "information* system*" OR "interact* health* communication applicatio*" OR "comput* application*" OR "comput* mediat* communication*" OR "therap* comput* assist*" OR "comput* assist*" OR "telecommunication* media" OR "health* record*" OR "online service*" OR "online therap*" OR internet OR "web-based" OR website

3. selfmanag* OR "self-manag*" OR selfcar* OR "self-car*" OR selftreat* OR "self-treat*" OR selfregulat* OR "self-regulat*" OR selfhelp* OR "self-help*" OR selfmonitor* OR "self-monitor*" OR selfmedicat* OR "self-medicat*" OR "disease manag*" OR "disease controllability"

4. "cardi* disease*" OR "cardi* disorder*" OR "heart disease*" OR "heart failure" OR "cardi* failure" OR “hypertension” OR “atrial fibrillation” OR ”coronary artery disease” OR “peripheral artery disease”

5. #1 AND #2 AND #3 AND #4

### ACM Digital Library: Searched in ‘The ACM Guide to Computing Literature’ by ‘any field’ and filtered by >= 2008 publication year. Set 2 (eHealth related terms) removed due to technological specificity of database.

1. Input to Edit Query: ("self-management" "self-care" "self-treatment" "self-regulation" "self-help" "self-monitoring" "self-medication" "disease management" "disease controllability") AND (framework theory model "participatory design" "persuasive design" "user centred" "user centered" "human centred" "human centered" "business model") AND ("cardiovascular disease" "cardiovascular disorder" "heart disease" "heart failure" "cardiac failure" "hypertension" "atrial fibrillation" "coronary artery disease" "peripheral artery disease")

2. Apply filter: “Published since” 2008

3. Full query syntax

"query": { ("self-management" "self-care" "self-treatment" "self-regulation" "self-help" "self-monitoring" "self-medication" "disease management" "disease controllability") AND (framework theory model "participatory design" "persuasive design" "user centred" "user centered" "human centred" "human centered" "business model") AND ("cardiovascular disease" "cardiovascular disorder" "heart disease" "heart failure" "cardiac failure" "hypertension" "atrial fibrillation" "coronary artery disease" "peripheral artery disease") }

"filter": {"publicationYear":{ "gte":2008 }},

{owners.owner=GUIDE}

### Cochrane Library: Searched by ‘Title, Abstract, Keywords’ and search limit of ‘Publication year’ from 2008.

1. fram* OR theor* OR model* OR "participatory" OR "persuasive" OR "user-cent*" OR "human-cent*" OR "business model*"

2. ehealth* OR "e-health*" OR "electronic health*" OR mhealth* OR "m-health*" OR "mobil* health*" OR "mobil* application*" OR "mobil* intervention*" OR telehealth* OR "tele-health*" OR telemedic* OR "tele-medic*" OR telecar* OR "tele-car*" OR telemonitor* OR "tele-monitor*" OR "health informatic*" OR "medic* informatic*" OR "informatic* intervention*" OR "*care informatic*" OR "personal* informatic*" OR "nurs* informatic*" OR "enabl* technolog*" OR "health interact* technolog*" OR "health informat* technolog*" OR "health* technolog*" OR "assist* health* technolog*" OR "health* assist* technolog*" OR "persuas* technolog*" OR "persuas* health* technolog*" OR "technology-supported" OR "behavio* intervention* technolog*" OR "digital* behavio* change intervention*" OR "digital* intervention*" OR "behavio* change support system*" OR "*care information* system*" OR "interact* health* system*" OR "decision* support* system*" OR "online system*" OR "comput* system*" OR "communication system*" OR "information* system*" OR "interact* health* communication applicatio*" OR "comput* application*" OR "comput* mediat* communication*" OR "therap* comput* assist*" OR "comput* assist*" OR "telecommunication* media" OR "health* record*" OR "online service*" OR "online therap*" OR internet OR "web-based" OR website

3. selfmanag* OR "self-manag*" OR selfcar* OR "self-car*" OR selftreat* OR "self-treat*" OR selfregulat* OR "self-regulat*" OR selfhelp* OR "self-help*" OR selfmonitor* OR "self-monitor*" OR selfmedicat* OR "self-medicat*" OR "disease manag*" OR "disease controllability"

4. "cardi* disease*" OR "cardi* disorder*" OR "heart disease*" OR "heart failure" OR "cardi* failure" OR "hypertension" OR "atrial fibrillation" OR "coronary artery disease" OR "peripheral artery disease"
